# Microbiota and transcriptome changes of *Culex pipiens pallens* larvae exposed to *Bacillus thuringiensis israelensis*

**DOI:** 10.1038/s41598-021-99733-8

**Published:** 2021-10-12

**Authors:** Ruiling Zhang, Wenjuan Liu, Qian Zhang, Xinyu Zhang, Zhong Zhang

**Affiliations:** 1 Collaborative Innovation Center for the Origin and Control of Emerging Infectious Diseases, Shandong First Medical University (Shandong Academy of Medical Sciences), Taian, China; 2 School of Basic Medical Science, Shandong First Medical University, Taian, China

**Keywords:** Entomology, Health care, Public health

## Abstract

*Culex pipiens pallens* is an important vector of lymphatic filariasis and epidemic encephalitis. Mosquito control is the main strategy used for the prevention of mosquito-borne diseases. *Bacillus thuringiensis israelensis* (*Bti*) is an entomopathogenic bacterium widely used in mosquito control. In this study, we profiled the microbiota and transcriptional response of the larvae of *Cx. pipiens pallens* exposed to different concentrations of *Bti*. The results demonstrated that *Bti* induced a significant effect on both the microbiota and gene expression of *Cx. pipiens pallens*. Compared to the control group, the predominant bacteria changed from Actinobacteria to Firmicutes, and with increase in the concentration of *Bti*, the abundance of Actinobacteria was gradually reduced. Similar changes were also detected at the genus level, where *Bacillus* replaced *Microbacterium*, becoming the predominant genus in *Bti*-exposed groups. Furthermore, alpha diversity analysis indicated that *Bti* exposure changed the diversity of the microbota, possibly because the dysbiosis caused by the *Bti* infection inhibits some bacteria and provides opportunities to other opportunistic taxa. Pathway analysis revealed significant enhancement for processes associated with sphingolipid metabolism, glutathione metabolism and glycerophospholipid metabolism between all *Bti-*exposed groups and control group. Additionally, genes associated with the Toll and Imd signaling pathway were found to be notably upregulated. *Bti* infection significantly changed the bacterial community of larvae of *Cx. pipiens pallens*.

## Introduction

Mosquitoes cause enormous public health risks by transmitting various infectious agents of diseases^1^. More than one-half of the population of the world is threatened by mosquito-borne diseases^2–4^, causing a huge burden to human health and the economy^5^. *Culex pipiens pallens* is one of the sibling species of the *Culex pipiens* complex and the most prevalent *Culex* species in northern China. As the primary vector of lymphatic filariasis, epidemic encephalitis and other diseases, this mosquito poses a serious threat to human health^6–8^. Due to the lack of effective vaccines and increasing drug resistance in pathogens, vector control is an important tool for the prevention of major mosquito-borne diseases worldwide^9,10^. As a rapid, convenient and effective method of controlling mosquitoes, chemical insecticide is a core component for mosquito-borne disease control programs and has significantly reduced the prevalence of vector-borne diseases around the world^11^. However, the long-term large-scale use of chemical insecticides can result in many deleterious effects, including selection for insect resistance to pesticides, morbidity of non-target insects, and devastating effects on the environment and ecosystems^12–15^, which have become a major obstacle to the control of mosquito-borne diseases.

*Bacillus thuringiensis* (*Bt*) is an entomopathogenic bacterium widely used in biocontrol of insect pests. Extensive studies have revealed that the toxicity of *Bt* is induced by parasporal crystalline proteins protoxins synthesized during sporulation. When activated by proteolytic cleavage in the midgut after ingestion by target larvae, the active toxins bind to midgut receptors and ultimately form pores that destabilize osmotic balance if cells of the midgut lining, leading to death of the host^16,17^. The high level of specificity of *Bt* subspecies toward insects has also been proposed to be associated with microbiota of its hosts^18,19^. Some studies revealed that host-associated microbiota might be required for effective *Bt* killing of its insect host^20^. Others found that a bacteriocin produced by *Bt* can inhibit the growth of some bacteria^21^.

Hundreds of *Bt* subspecies have been described to date, and each of these subspecies produces a specific set of toxins as a crystal during sporulation, and the crystals have been successfully used for the control of agricultural pests and medical vectors^21,22^. Among all of the *Bt* subspecies, parasporal crystalline proteins generated by the bacterium *B. thuringiensis israelensis* (*Bti*) are toxic when ingested by mosquitoes. The World Health Organization (WHO) has proposed the use of *Bti* for the prevention and control of arboviral diseases^23^. As a powerful and environmentally friendly biological insecticide^24,25^, *Bti* has been widely used since 1976 and exhibits a specific biolarvicidal effect for controlling species of the Culicidae family^26–28^. Although the action of *Bti* has been extensively studied, the underlying mechanisms and relationships with microbiota of hosts are far from being resolved. The microbiota of the mosquito is highly variable, is shaped by many factors such as environment and life history traits^29–32^ and can actively participate in defense against pathogens^33–35^. It is important to discriminate changes in the microbial community, which might be helpful in revealing the association of microbiota with *Bti* toxicity. In addition, differentially expressed genes and molecular pathways induced by *Bti* infection might contribute to better understanding of the molecular regulation of the mosquito response to *Bti* and help in evaluating potential targets for molecular-based vector control. In the present study, which is based on the 16S rRNA gene and RNA sequencing (RNA-Seq), we explored the dynamic changes in the microbiota of *Cx*. *pipiens pallens* that are exposed to different *Bti* concentrations and identified the differentially expressed genes triggered by the *Bti* infection.

## Material and methods

### Mosquito rearing

*Culex pipiens pallens* colony used in this study were collected from Shandong Province and maintained in the laboratory for years. Mosquitoes were maintained in a rearing room at 27 ± 1 °C and 65% relative humidity (RH) with a daily photoperiod of 14:10 h (L:D). The larvae were reared in dechlorinated tap water in plastic containers and fed with a slurry of pork liver powder (homemade) and yeast mixed with distilled water. Pupae were collected and placed into a beaker and then reared in insect cages (35 × 35 × 35 cm) until adults emerged. Newly emerged adults were provided with 10% sugar solution ad libitum. Adult females of 4–5 days old were supplied a diet of mouse blood. Mouse was fixed on a plastic board and put into the insect cages. Experimental protocol for all animal experiments was in accordance with guidelines and with permission from the local animal ethics committee and with permission from the Institutional Animal Care and Use Committee of Shandong First Medical University. The study was carried out in compliance with the ARRIVE guidelines^36^. Fully engorged females laid eggs in oviposition cups, and the eggs were transferred into plastic containers with dechlorinated tap water. All samples were flash frozen in liquid nitrogen immediately following collection and then stored at − 80 °C until for subsequent uses.

### *Bti* larvicide

The larvicide used in this study was liquid suspension of *Bacillus thuringiensis israelensis* (Lukang, Shandong, China). The concentrations used were referred to manufacturer’s introduction (2.5 ml larvicide/97.5 ml water). Application concentrations were set to Group A (0.5 ml larvicide/99.5 ml water), Group B (1 ml larvicide/99 ml water), Group C (1.5 ml larvicide/98.5 ml water) and Group D (100 ml water) as control group. Two hundred eggs of *Cx*. *pipiens pallens* were put into each treatment (Group A, B, C and D), respectively.

The larvicidal activity of different *Bti* concentrations was performed on third instar larvae. Twenty larvae were placed in 100 ml beaker that containing appropriate concentration of *Bti* solutions that corresponding to Group A, B, C and D, respectively. Mortality was assessed after 24 h, during which larvae were fed to avoid the influences caused by starvation. The assays were done in five replicates. The percentage of larval mortality was calculated for each concentration using Abbott’s formula^37^. In addition, dilutions of the stock were cultured on Potato Dextrose Agar (PDA) medium to evaluate spore counts of each *Bti* concentration.

### DNA extraction and 16S rRNA library preparation

Fifteen fourth instar larvae were randomly collected from each group (with three biological replicates for each group). All samples were surface sterilized with 75% ethanol for 5 s and then washed with sterile water for three times. Genomic DNA was extracted from the whole body of mosquitoes using DNeasy Blood and Tissue Kit (QIAGEN, United States) following the manufacturer’s instructions. DNA concentration and purity were monitored on 1% agarose gels. Each DNA sample was diluted to 1 ng/μl using sterile water. The V4 hypervariable region of the 16S rRNA gene was amplified using the primers 515F (5′-TCGTCGGCAGCGTCAGATGTGTATAAGAGACAGGTGCCAGCMGCCGCGGTAA-3′) and 806R (5′-GTCTCGTGGGCTCGGAGATGTGTATAAGAGACAGGGACTACHVGGGTWTCTAAT-3′). All PCR reactions were carried out with 15 μl of PCR Master Mix (New England Biolabs), including 0.2 μM of primers and 10 ng DNA template. Thermal cycling consisted of the initial denaturation for 1 min (98 °C), followed by 30 cycles of denaturation at 98 °C for 10 s, annealing at 50 °C for 30 s, and elongation at 72 °C for 30 s with final extension for 5 min (72 °C). PCR products were detected in 2% agarose gel and then purified with the Qiagen Gel Extraction Kit (Qiagen, Germany). Sequencing libraries were generated using a DNA PCR-Free sample preparation kit (Illumina, USA) following the manufacturer's recommendations. The quality of the library was assessed on the Qubit 2.0 Fluorometer (Thermo Scientific) and Agilent Bioanalyzer 2100 system. The resulting libraries were sequenced on an Illumina NovaSeq6000 platform.

### 16S rRNA gene sequence data analysis

Paired-end 16S reads were merged using FLASH V1.2.7^38^. QIIME 2 (release 2018.8)^39^ was used to filter the raw reads and obtain high-quality clean reads. Chimera sequences were identified and removed by compared clean tags with the Silva database (https://www.arb-silva.de/)^40^ using the UCHIME algorithm^41^. Uparse v7.0.1001^42^ was used to perform sequences analysis, and sequences with ≥ 97% similarity were assigned to the same OTUs. The taxonomy of a representative sequence of each OTU was annotated by comparison to the Silva Database based on the mothur algorithm. Taxonomy summaries of relative abundance data at different taxonomic levels were generated.

Alpha (within-sample) and beta (between-sample) diversity was calculated with QIIME 2 and displayed with R software V 2.15.3. Chao1 (richness estimators) measures diversity based on only the number of OTUs present, whereas Shannon (diversity indices) considers both abundance and evenness of the OTUs in a community. Good’s Coverage was used to provide a description of rarefaction in terms of the total sampled OTU diversity^43^. Overlapping and core microbiota of each sample were shown in Venn diagrams, which were generated by use of a web tool (http://bioinformatics.psb.ugent.be/webtools/Venn/). Principal coordinate analysis (PCoA) was performed to obtain principal coordinates and visualize complex multidimensional data.

### RNA extraction, library construction and sequencing

Total RNA was extracted from 15 fourth instar larvae that were randomly selected from each *Bti* concentration group and control group (with three biological replicates for each group) using the RNAiso plus reagent (TaKaRa, Japan). RNA degradation and contamination were checked with denaturing agarose gel electrophoresis, and a NanoDrop (ND-1000) spectrophotometer (NanoDrop, USA) was used to test the quality and quantity of the purified RNA. The integrity of RNA was assessed by 1.5% agarose gel electrophoresis.

Transcriptome sequencing libraries were generated using an RNA Library Prep Kit for Illumina (NEB, USA) according to the manufacturer’s instructions. mRNA was purified from total RNA using poly-T oligo-attached magnetic beads and was fragmented into short fragments using divalent cations under elevated temperature in NEBNext First Strand Synthesis Reaction Buffer (5X). First strand cDNA was synthesized using random hexamer primers and mRNA templates. Second strand cDNA synthesis was subsequently synthesized using DNA polymerase I and RNase H, dNTPs and buffer. After end reparation and adenylation of the 3′ ends, short fragments were connected using the NEBNext Adaptor with a hairpin loop structure. The library fragments were purified with an AMPure XP system (Beckman Coulter, Beverly, USA) for selection of suitable fragments after PCR amplification. Finally, the PCR products were purified (AMPure XP system) and the library quality was assessed on a Bioanalyzer 2100 (Agilent, USA). High-throughput transcriptome sequencing was performed on an Illumina NovaSeq platform.

### RNA-Seq data analysis

Firstly, the quality and number of the reads of each sample were assessed using FastQC v 0.11.4^44^. Fastq files were trimmed using Trimmomatic v 0.38^45^. Clean data (clean reads) were obtained by removing adapter, poly-N and low-quality reads from the raw data. Then, the Q30 and GC content was calculated based on the clean data. The paired-end clean reads were aligned to the reference genome using Hisat2 v2.1.0^46^. The genome sequences of *Cx. pipiens pallens* (PRJNA702155) were chosen as reference genes. The reads of each sample mapped to reference genes were assembled by StringTie v1.3.3b^47^. Then, the fragments Per Kilobase of the transcript sequence per millions mapped reads (FPKM) base pairs of each gene were calculated based on the length of the gene and read counts mapped to this gene.

Differential expression analysis of samples was performed using the DESeq2 R package (1.16.1). *P* values of the results were adjusted using the Benjamini and Hochberg’s approach for controlling the false discovery rate. Genes with an adjusted *P* value < 0.05 were assigned as differentially expressed genes (DEGs). Kyoto Encyclopedia of Genes and Genomes (KEGG) pathway and Gene Ontology (GO) enrichment analyses of differentially expressed genes was implemented by the clusterProfiler R package, in which gene length bias was corrected. GO terms and KEGG pathways with corrected *P* value less than 0.05 were considered significantly enriched in differential expressed genes.

## Results

### Microbiota diversity

**Figure 1:**
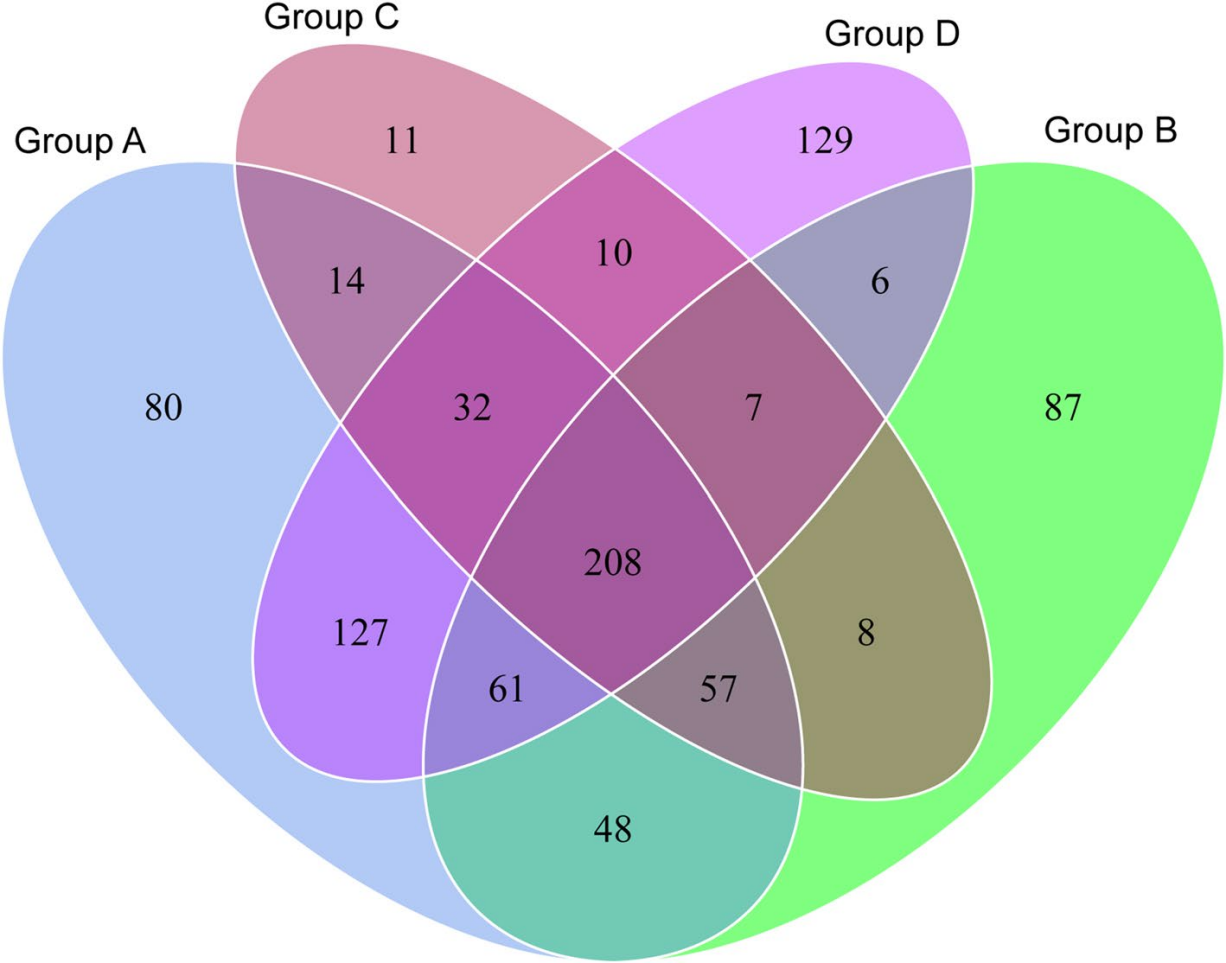
Venn diagram showing the number of OTUs shared between four groups.

**Figure 2:**
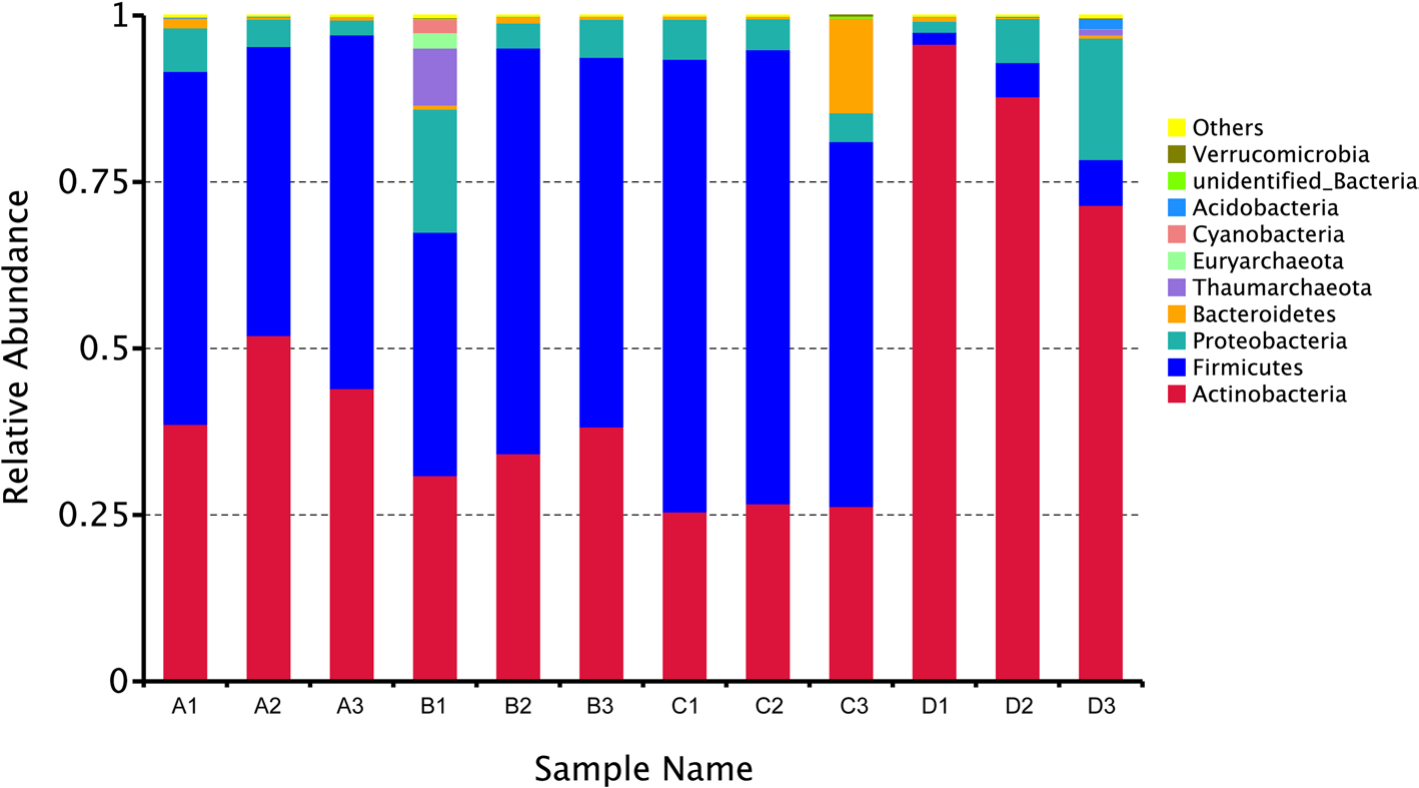
Bar plot of the relative abundances of microbial community at the rank of phyla level.

A total of 1,064,820 16S rRNA gene sequences were generated, 80,377 to 99,826 reads from different samples (SRA: PRJNA675318). After quality control, 778,775 reads were used for the following analysis and clustered into 886 OTUs, including 844 bacteria, 32 archaea and 10 unclassified. Together, the bacterial OTUs were associated with 20 unique phyla, 34 classes, 79 orders, 144 families, 252 genera and 158 species. There were 208 shared OTUs among the four experimental groups, and 80, 87, 11 and 129 unique OTUs were detected in each group, respectively (Fig. 1, Table S1). Firmicutes was the dominant phylum of mosquito samples exposed to *Bti*, while the predominant phylum in the control groups was Actinobacteria (Fig. 2). The abundance of Actinobacteria decreased gradually with increasing *Bti* concentrations (Fig. 2). Similarly, this tendency was also detected at the genus level. *Microbacterium* was the predominant genus in the control group. However, the abundance of *Microbacterium* was significantly reduced in all *Bti*-exposed groups, and the abundance was lower with an increase in *Bti* concentration. In addition, *Bacillus* became the dominant genus in all *Bti*-exposed groups (Fig. S1).

**Figure 3:**
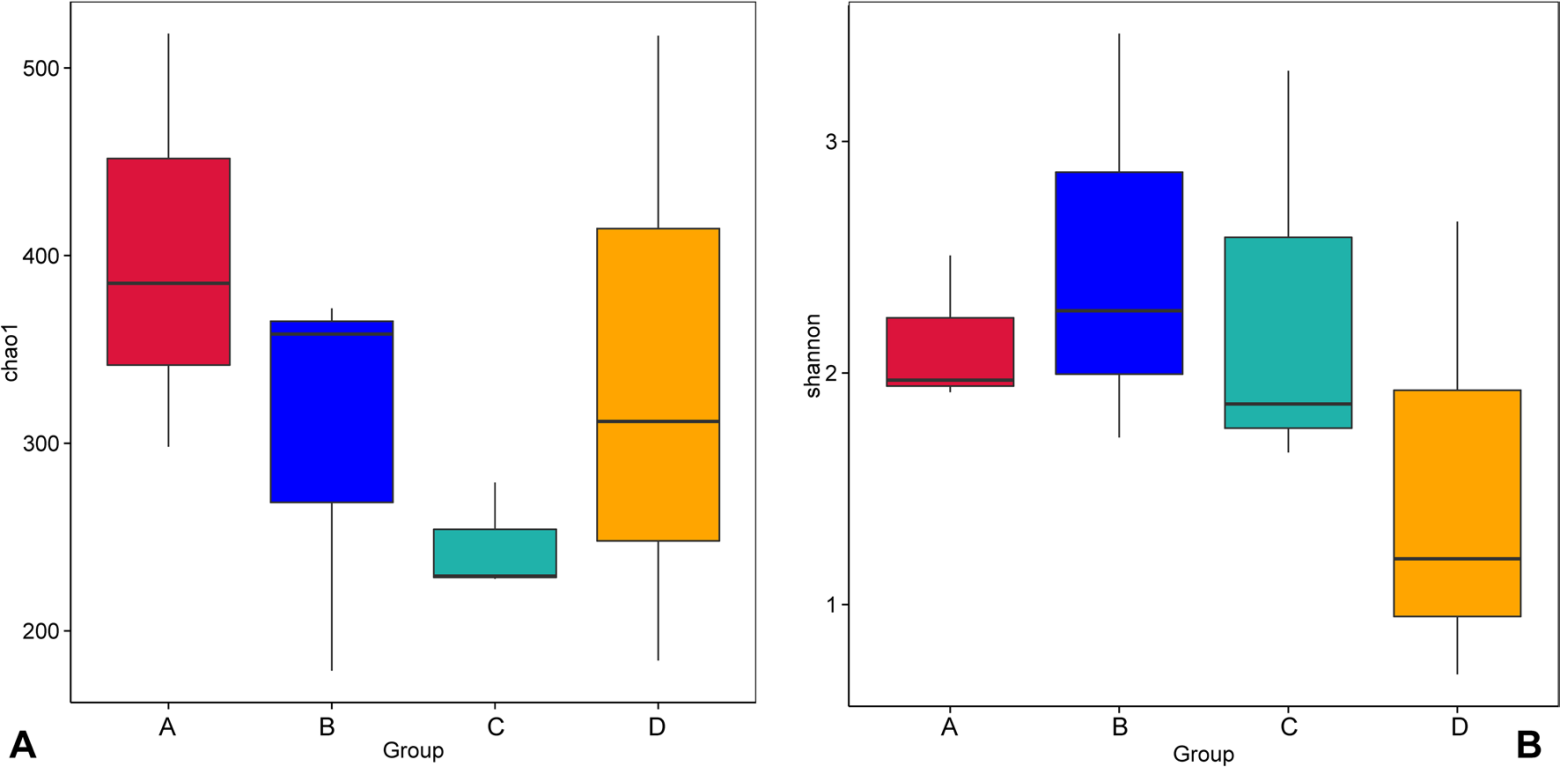
Boxplot representation of chao1 (**A**) and Shannon (**B**) indexes.

**Figure 4:**
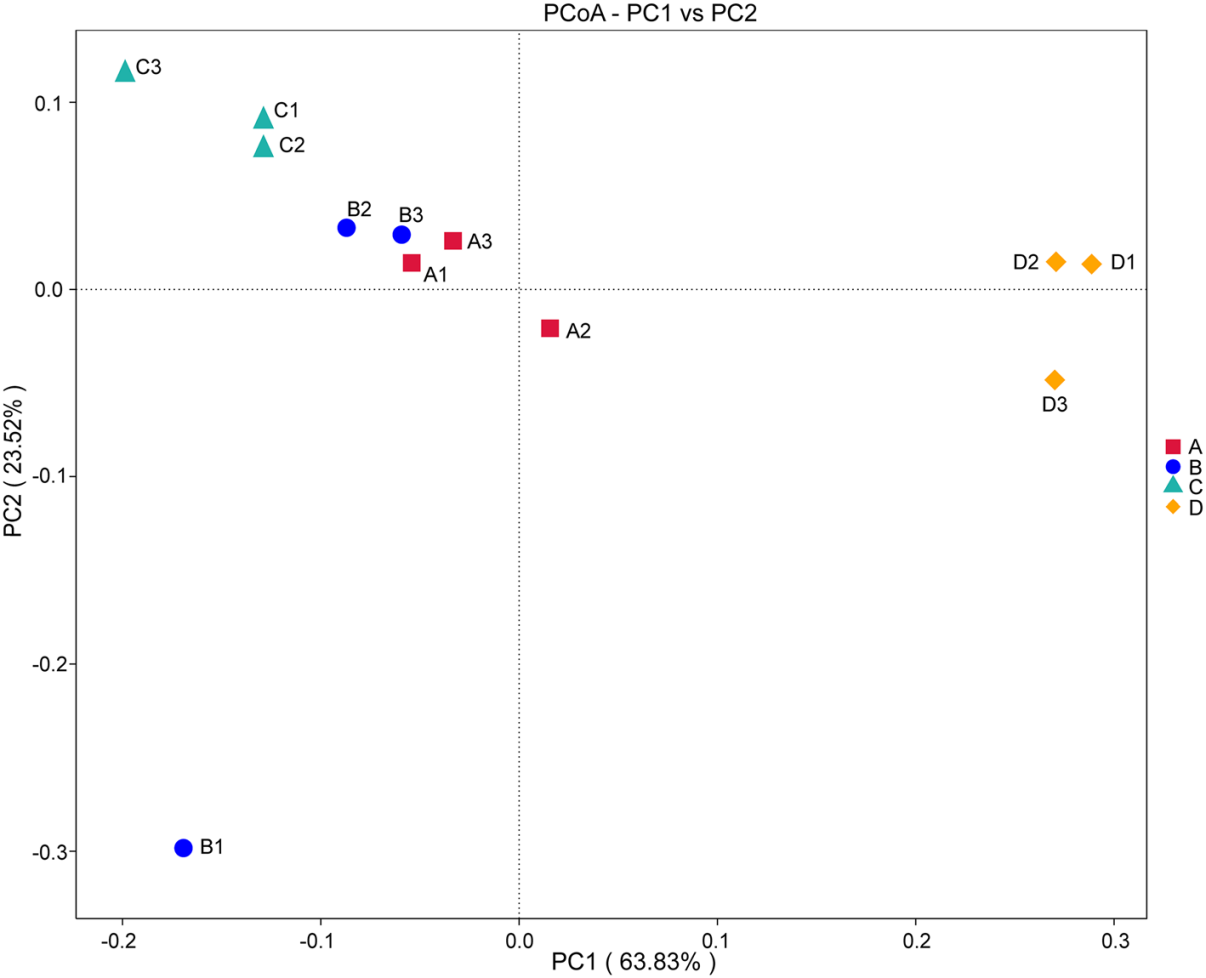
Principal coordinate analysis (PCoA) of the microbial communities from different groups.

Good's coverage ranged from 99.9 to 100% for each sample, suggesting that the identified 16S rDNA sequences were sufficient to fully represent the bacterial diversity of the samples in this study (Table 1). Consistent with the decreased trend in LC_50_ and LC_90_ values in Group A, B, and C (Table S2), the Chao1 index decreased with increasing *Bti* concentrations, indicating that high concentrations of *Bti* reduced the bacterial richness of *Cx. pipiens pallens*. Furthermore, the Chao1 index revealed that the bacterial richness of the control group was higher than group C. Both group A and B had higher microbial richness than the control group (Fig. 3A). Comparison of the Shannon index between groups exposed to *Bti* with the control group demonstrated a significant increase in the diversity in the *Bti*-exposed groups (Fig. 3B). The structure of bacterial communities between groups was evaluated with PCoA analysis. The weighted UniFrac PCoA showed that 63.83% (axis 1) and 23.52% (axis 2) of the bacterial composition variation among the four groups were explained (Fig. 4). All samples exposed to *Bti* clustered separately from the control group, indicating that the microbiota of *Cx*. *pipiens pallens* was significantly changed upon exposure to *Bti*. Furthermore, the samples exposed to different *Bti* concentrations clustered separately, conforming that the different bacterial communities exhibit a pattern of specialization (Fig. 4).

**Table 1:** Details of alpha diversity indexes of four groups.

Bti concentration	Sample name	Observed species	Shannon	Chao1	Goods coverage
3 ITU	A1	484	2.497	518.348	0.999
	A2	321	1.91	385.208	0.999
	A3	250	1.962	298.158	0.999
9 ITU	B1	338	3.45	358.128	0.999
	B2	330	2.26	371.902	0.999
	B3	155	1.715	178.786	0.999
15 ITU	C1	205	1.859	229.241	0.999
	C2	252	1.651	279.051	0.999
	C3	203	3.291	227.7	0.999
0 ITU	D1	175	0.698	184.255	0.999
	D2	274	1.194	311.652	0.999
	D3	459	2.643	517.184	0.999

### RNA-seq

**Figure 5:**
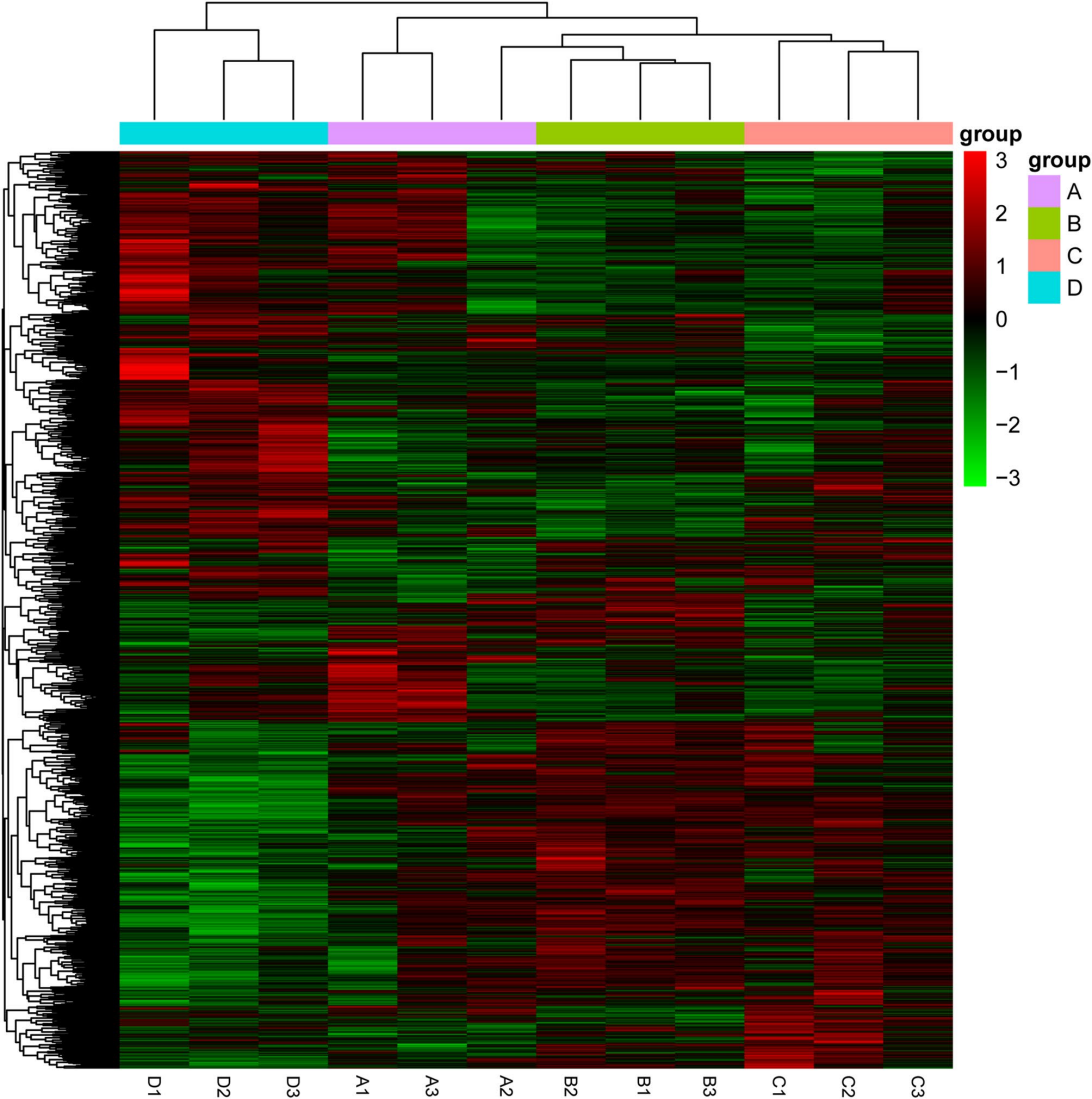
Heatmap showing the expression levels of differentially expressed genes (DEGs) in four groups.

**Figure 6:**
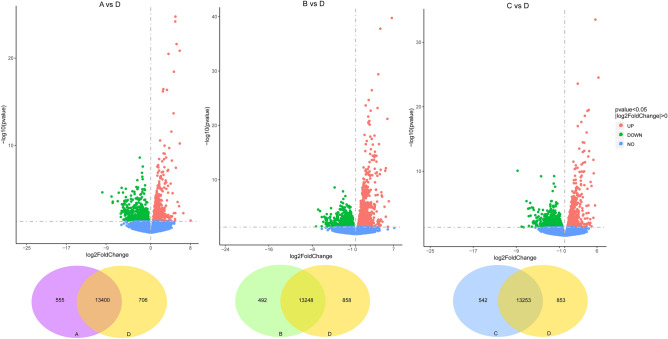
Scatter diagrams exhibiting differentially expressed gene profiling comparisons between group D and other three *Bti* exposed groups. A, Group A vs Group D; B, Group B vs Group D; C, Group C vs Group D. Blue point, non-significant difference in gene; red point: up-regulated gene; green point: down-regulated gene.

All RNA-seq data have been deposited at SRA (NCBI) with the accession number PRJNA675746. Altogether, 18,078 genes were identified, and 15,959 genes were annotated according to the referenced genome. To test the effect of *Bti* on *Cx. pipiens pallens* gene regulations, DEG analyses were carried out for groups exposed to *Bti* compared to their expression in the control group, and between groups exposed to different *Bti* concentrations. To gain an initial overview of the data, heatmaps of all the samples were generated (Fig. 5). A major variation in color pattern was visually identifiable between the control group and the groups exposed to *Bti* (Fig. 5). A total of 1,208 genes were identified as differentially expressed between Group A and D, among which 566 genes were upregulated, and 1,336 genes were downregulated (Table 2; Fig. 6). When the gene expression levels were compared between Group B and D, 2,277 genes were differentially expressed, 1,251 genes and 1,026 genes were upregulated and downregulated, respectively (Table 2; Fig. 6). A total of 1,538 differentially expressed genes were found between Group C and D, including 783 upregulated genes and 755 downregulated genes (Table 2; Fig. 6). In contrast, there were substantially fewer differentially expressed genes in Groups A, B and C, suggesting fewer changes at the molecular level in these groups (Table 2).

**Table 2:** Number of differentially expressed genes between different groups.

Groups	All	Up	Down
A vs D	1208	566	642
B vs D	2277	1251	1026
C vs D	1538	783	755
A vs B	784	324	460
A vs C	726	441	285
B vs C	542	318	224

The threshold value of significance was DESeq2 p value ≤ 0.05 |log2FoldChange|≥ 0.0.

**Figure 7:**
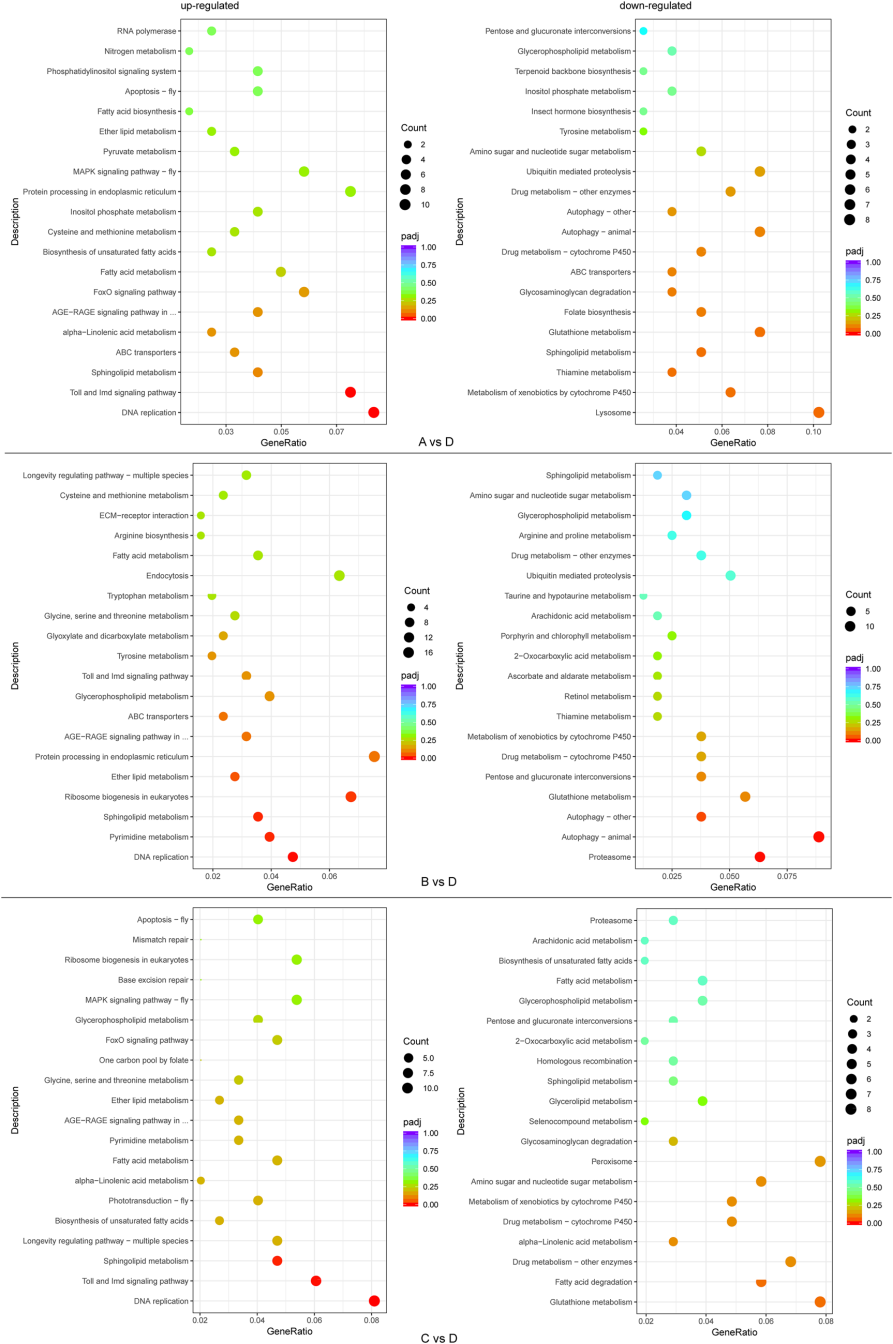
KEGG pathway enrichment analysis of up- and down-regulated DEGs.

The potential function of each DEGs was explored using GO and KEGG analyses. In the GO classification, the DEGs in all comparisons (A vs B, A vs C, A vs D, B vs C, B vs D, C vs D) were divided into three main categories, including biological processes, cellular components, and molecular functions. The enriched functional terms of the DEGs demonstrated that molecular function was the most varied cluster between the groups exposed to *Bti* and the control group (Fig. S2). In the KEGG analysis, the top 20 pathways were included in the subsequent analysis, and the metabolism pathways were the most enriched, followed by genetic information processing, environmental information processing, cellular processes, organismal systems, and human diseases (Table S3). ‘Sphingolipid metabolism’, ‘glutathione metabolism’ and ‘glycerophospholipid metabolism’ were three metabolism pathways presented in all comparison groups (A vs D, B vs D, C vs D); and ‘DNA replication’ of genetic information processing, ‘ABC transporters’ of environmental information processing, ‘Toll and imd signaling pathway’ of organismal systems were also found in three comparison groups. Furthermore, ‘drug metabolism’ of the metabolism pathway was detected only in B vs D and C vs D comparisons; ‘autophagy’ of cellular processes pathway was involved in the A vs D and B vs D groups, while ‘apoptosis’ was found in the C vs D groups (Fig. 7). Additionally, the KEGG pathway enrichment analysis demonstrated that the ‘lysosome’, ‘autophagy’ and ‘glutathione metabolism’ pathway were significantly enriched for the downregulated genes corresponding to the A vs D, B vs D, C vs D groups, respectively. Furthermore, the ‘DNA replication’ pathway was significantly enriched in upregulated genes in all three comparison groups (Fig. 7).

Compared with the control group, the genes in the drug metabolism (cytochrome P450) pathway were significantly downregulated in all three groups exposed to *Bti* (Table S4). A large number of Toll and imd signaling pathway genes were upregulated in the three comparison groups (A vs D, B vs D, C vs D). Only three genes (6044929, 6052047, and novel.497) were found to be downregulated. The autophagy pathway involved 10 upregulated genes and 14 downregulated genes (Table S4).

## Discussion

Mosquito-borne diseases constitute a major burden for public health problems worldwide^10,48,49^.

The application of *Bti* to kill mosquito larvae is an effective strategy for mosquito control and safety for the environment^33,50,51^. However, the underlying mechanism of *Bti* to kill mosquitoes has thus far not been completely elucidated.

Following the infection process of *Bti*, both the insect immune system and microbiota were changed. The activated immune response and dysbiosis of the microbiota have an important effect on the pathogenicity of *Bti*^52^. A better understanding of the abundance and compositional changes of bacteria could potentially be helpful to illuminating the interaction between *Bti* toxicity and microbiota, exploring available bacterial taxa strengthening *Bti* toxicity to mosquitoes, which finally contributes to the development of new sustainable mosquito control strategies. In the current study, the predominant bacteria of all *Cx. pipiens pallens* larvae exposed to *Bti* was changed from Actinobacteria to Firmicutes, and the abundance of Actinobacteria was decreased with increase in the *Bti* concentrations (Fig. 2). Results of alpha diversity analysis suggested that Chao1 indexes of Group A and B were higher than Chao1 indexes of Group D, while Group C was lower than Group D; Shannon indexes of all groups exposed to *Bti* were exceeded Shannon indexes of Group D. Previous studies have proved that *Bt* can inhibit the growth of bacteria by producing bacteriocins^28^, which are extracellular peptides or proteins with bactericidal and bacteriolytic effects. For instance, cell wall hydrolyzing enzymes produced by bacteriocins, nisin and Pep5, can cause rapid lysis of staphylococcal^53,54^. In addition, some bacteria can also degrade toxins of *Bt*^33,55,56^. Based on the present results, we can deduce that *Bti* might cause imbalance of the microbiota of *Cx. pipiens pallens* and reduced abundance of Actinobacteria, while some opportunistic bacteria, like Firmicutes, grow rapidly to render the increase of bacterial diversity and changed abundance of bacteria of larvae infected with *Bti*. However, due to the competition between *Bti* and microbiota, high toxicity of *Bti* finally causes reduction of bacteria richness.

In addition to the competitive relationships between *Bti* and microbiota, beneficial interactions were also reported. Insects exhibited increased tolerance to *Bti* toxicity after removal of gut bacteria with antibiotics^20,57,58^. Bacteria of *Helicoverpa armigera*, a lepidopteran pest, have been proven produce proteases that contribute to the activation of *Bti* protoxins into toxins^59^. Reasons for the rapid increase of Firmicutes in samples exposed to *Bti* are worthy of further investigation to determine whether these bacteria play roles similar to their roles in *H*. *armigera* or whether they can degrade *Bti* toxins and guarantee that mosquitoes survive despite dysbiosis. Results of PCoA analysis demonstrated that microbiota samples exposed to *Bti* clustered separately from the control group, and the samples exposed to different concentrations of *Bti* clustered separately (Fig. 4). Therefore, we can conclude that *Bti* exposure changes the bacterial composition of *Cx. pipiens pallens* and the microbiota characteristics are determined by the *Bti* concentration which they were infected.

In addition to the investigation of the composition of the microbiota, gene expression profiles were also explored. Comparisons between *Bti*-exposed groups and control groups revealed that *Bti* induced a significant effect on the gene expression of *Cx. pipiens pallens* larvae. Overall, there was no obvious difference in gene expression among groups exposed to different concentrations of *Bti*, while more than 1200 DEGs were identified as differentially expressed when comparing each of the *Bti*-exposed groups with the control group (Table 2). The pathway analysis revealed a significant enrichment of genes in processes associated with sphingolipid metabolism, glutathione metabolism and glycerophospholipid metabolism, which were found in all the comparison groups (A vs D, B vs D, C vs D). These three pathways are usually involved in forming of membranes and helping host defense against pathogens^60,61^. The overrepresentation of these pathways might reflect the damage caused by *Bti* and the defense reaction that was activated against this. Consistent with these results, we also found enriched genes associated with the Toll and imd signaling pathway, which are two major immune pathways critical for the immune response that functions through the production of antimicrobial peptides (AMPs). AMPs are among the important members involved in humoral immune responses of insects^52^. The pronounced upregulation of gene expression associated with the Toll and imd signaling pathway indicated that larvae exposed to *Bti* mount a strong defense.

Though the killing mechanism of *Bti* has yet to be fully elucidated, its infections can cause tissue lesions and dysregulation of the gut environments for insects, and the microbiota residents in the host midgut trigger lethal septicemia^62,63^. Accordingly, we can hypothesize that the pathological damage and dysbiosis caused by *Bti* infection might activate protection mechanisms and lead to systematic variations in the expression of different genes. Therefore, the enrichment of drug metabolism (B vs D, C vs D groups), autophagy (A vs D, B vs D groups) and apoptosis (C vs D group) pathways in different comparison groups might reflect the differing extent of the damages and subsequent self-protection activity of the larvae, which are determined by the different levels of *Bti* toxicity to which they were exposed.

## Conclusions

Overall, in this study differences both in the microbiota and gene expression between samples exposed to different concentrations of *Bti* and control groups were observed. These data may constitute a starting point, which further suggests that bacteria play important roles in the immune reaction of mosquitoes. More studies are needed to reveal potential associations between the relative abundances of certain taxa in the microbiota, host gene expression levels and corresponding pathways. The identification of specific candidate bacteria and genes related to defense reactions might enhance our understanding of the interactions between *Bti* and mosquitoes, which will provide novel insights into the development of mosquito control strategies.

## Supplementary Information

Supplementary Information 1.

Supplementary Information 2.

Supplementary Information 3.

Supplementary Information 4.

Supplementary Information 5.

Supplementary Information 6.
