# Supplementary figures and images for: Microbiota and transcriptome changes of Culex pipiens pallens larvae exposed to Bacillus thuringiensis israelensis
Source: Sci Rep. 2021 Oct 12;11:20241. doi: 10.1038/s41598-021-99733-8 (PMC8511237; doi:10.1038/s41598-021-99733-8)

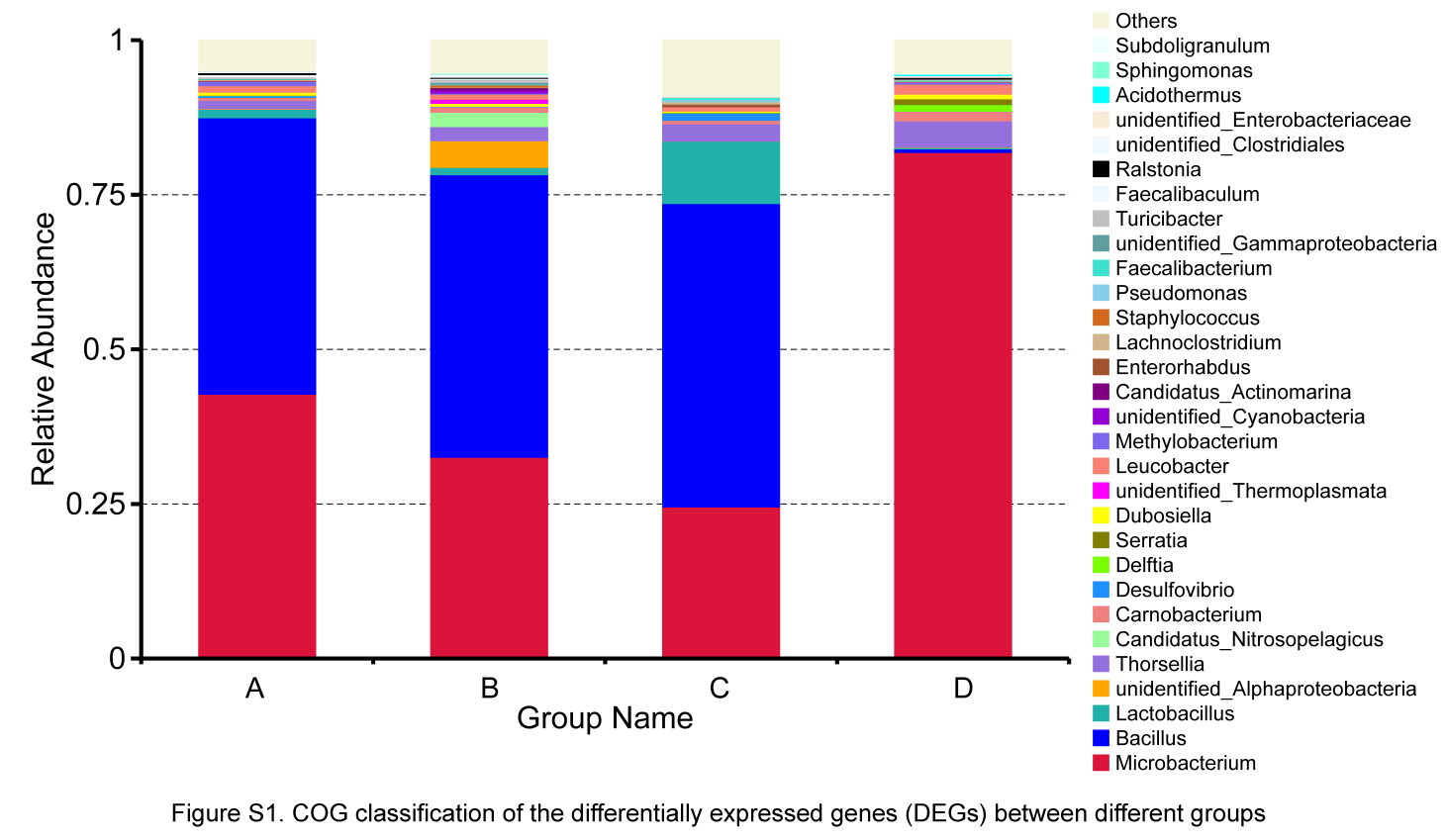

Supplement: Supplementary file 1 — Supplementary Information 1. [file 41598_2021_99733_MOESM1_ESM.tif]

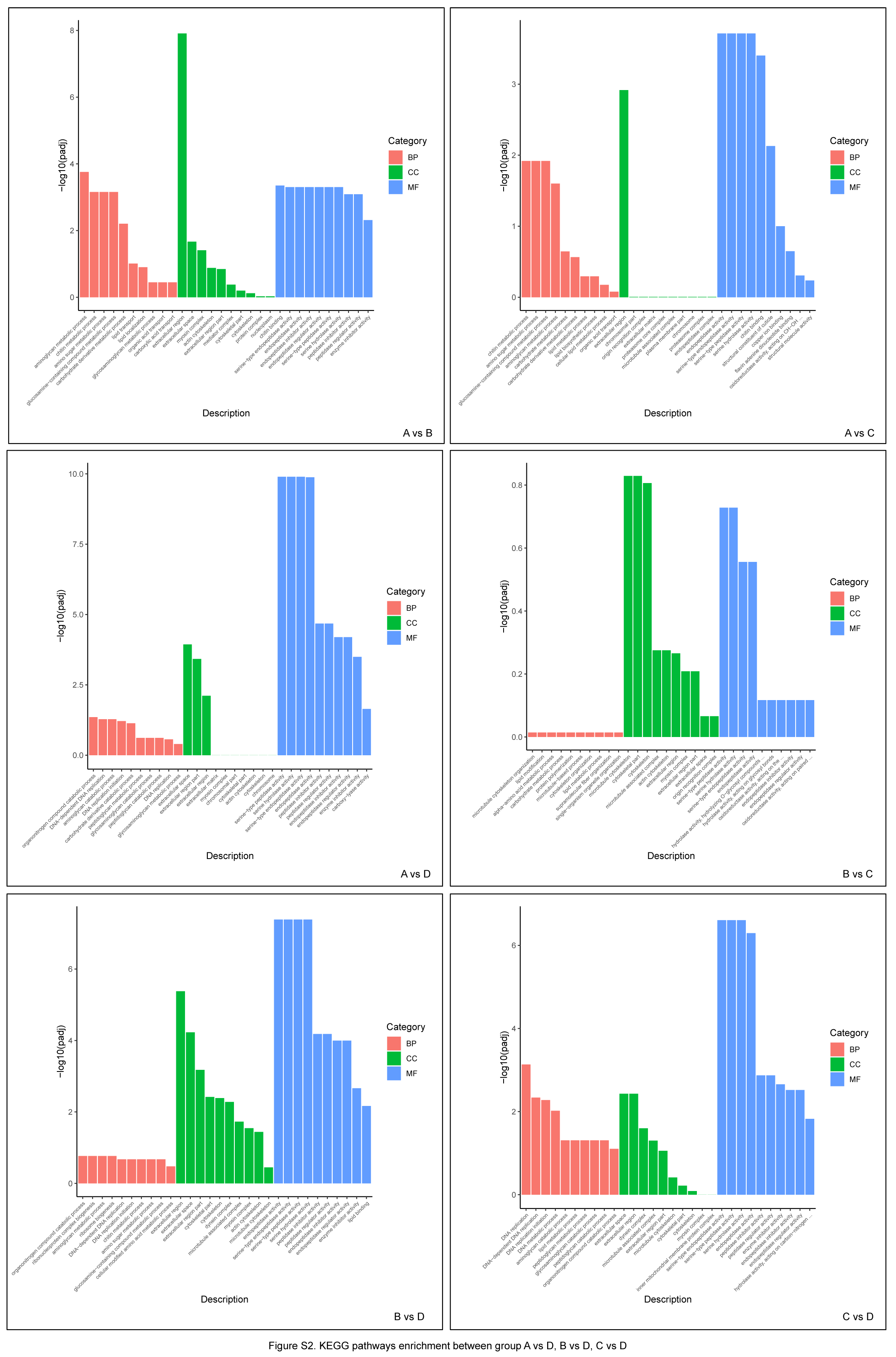

Supplement: Supplementary file 2 — Supplementary Information 2. [file 41598_2021_99733_MOESM2_ESM.tif]
