# Supplementary material for: Microbiota and transcriptome changes of Culex pipiens pallens larvae exposed to Bacillus thuringiensis israelensis
Source: Sci Rep. 2021 Oct 12;11:20241. doi: 10.1038/s41598-021-99733-8 (PMC8511237; doi:10.1038/s41598-021-99733-8)
Supplement: Supplementary file 4 — Supplementary Information 4. [file 41598_2021_99733_MOESM4_ESM.doc]

**Table S2. Larvicidal effect of different *Bti* concentrations**

| Treatment | LC_50_ (*μ*g/mL) ^a^ | LC_90_ (*μ*g/mL) | CFU/μg × 10^5 b^ |
| --- | --- | --- | --- |
| Group A | 22.5 (18.6–26.8) | 120.1 (75.8-216.5) | 8.5±0.6 |
| Group B | 16.2 (12.7-18.5) | 90.6 (59.5-143.2) | 0.92 ± 0.3 |
| Group C | 14.0 (10.2-16.7) | 85.0 (48.2-132.6) | 0.83 ± 0.4 |

^a^ number in the brackets indicate 95% confidence intervals;

^b^ CFU, colony forming units.
