# Supplementary material for: Microbiota and transcriptome changes of Culex pipiens pallens larvae exposed to Bacillus thuringiensis israelensis
Source: Sci Rep. 2021 Oct 12;11:20241. doi: 10.1038/s41598-021-99733-8 (PMC8511237; doi:10.1038/s41598-021-99733-8)
Supplement: Supplementary file 6 — Supplementary Information 6. [file 41598_2021_99733_MOESM6_ESM.docx]

**Table S4. Differentially expressed genes in different pathways.**

| **Pathways** | **Description** | **Groups** | **Gene ID** |
| --- | --- | --- | --- |
| Toll and Imd pathway | Up-regulated | A vs D | 6035104/6030875/6044826/6036321/6032181/6040802/6038645/6041351/6034142 |
|  |  | B vs D | 6030875/6036321/6044826/6035104/6032181/6041351/6040802/6038645 |
|  |  | C vs D | 6030875/6035104/6044826/6036321/6040802/6041351/6046952/6038645/6032181 |
|  | Down-regulated | A vs D | 6036449/novel.497 |
|  |  | B vs D | novel.497/6044929/6052047 |
|  |  | C vs D | 6036449/novel.497 |
| Autophagy | Up-regulated | A vs D | 6032181/6032532/6051906/6037927 |
|  |  | B vs D | 6032181/6037927/6032532/6037885/6041063/6035232/6039698/6052011 |
|  |  | C vs D | 6051906/6032181/6037927/6037885/6051901 |
|  | Down-regulated | A vs D | 6031553/6046657/6038998/6036998/6032310/6033062 |
|  |  | B vs D | 6036998/6033062/6038998/6036001/6048165/6046657/6035367/6046088/6038558/6031553/6053226/6051914/6032310/6053655 |
|  |  | C vs D | 6038998/6036998/6046657 |
| Drug metabolism - cytochrome P450 | Up-regulated | A vs D | 0 |
|  |  | B vs D | 0 |
|  |  | C vs D | 0 |
|  | Down-regulated | A vs D | 6047043/6048852/6047044/6052371 |
|  |  | B vs D | 6047043/6047041/6047044/6031178/6037722/6045701 |
|  |  | C vs D | 6047043/6052515/6048852/6047044/6047041 |
